# Mental health literacy in a diverse sample of undergraduate students: demographic, psychological, and academic correlates

**DOI:** 10.1186/s12889-020-09696-0

**Published:** 2020-11-13

**Authors:** Rona Miles, Laura Rabin, Anjali Krishnan, Evan Grandoit, Kamil Kloskowski

**Affiliations:** 1 Psychology Department, Brooklyn College of the City University of New York, 2900 Bedford Avenue, Brooklyn, NY 11210 USA; 2 Psychology Department, Northwestern University, Evanston, IL USA

**Keywords:** Mental health literacy, Knowledge of mental health, Mental health, College students, Undergraduates, Discriminant Correspondence Analysis

## Abstract

**Background:**

Investigating variables associated with mental health literacy in the college-age population takes us one step closer to providing intervention for this vulnerable group, where growing rates of psychological disorders are a serious public concern. This study adds to the existing literature by incorporating, within a single model, multi-faceted variables (demographic, psychological, and academic) that contribute to mental health literacy in demographically and ethnically diverse college students.

**Methods:**

Participants were undergraduate students enrolled at nine different colleges that are part of a large, urban, public university system. A total of 1213 respondents (62.0% female, 73.3% non-white) completed an in-person assessment of mental health literacy and answered questions about demographics, college experience, and mental health experience. Data were analyzed to identify which variables best discriminated between high, mid-level, and low performers on this assessment.

**Results:**

Discriminant correspondence analysis revealed that the difference between high and low performers (accounting for 90.27% of the total variance) was driven by participants who had taken at least one course related to clinical psychology and who typically majored in psychology and applied health science fields. These participants were more likely to report being white, female, between the ages of 28–32, and in the fourth year or later of their undergraduate program. In addition, high performers were more likely to have been diagnosed and/or treated for a psychological disorder, have more experience with psychological disorders through personal, family, or peer history, and have families who are open to discussing mental health issues.

**Conclusion:**

The main contributor to variation in mental health literacy scores was having taken a clinical psychology course, followed by majoring in psychology. Importantly, our findings identified not only the high performers, but also the low performers, for whom an increase in knowledge and awareness of mental health is crucial to overall psychological well-being. These results have important implications for the design of educational interventions aimed at improving mental health literacy at the college level, especially for students who otherwise would not have been exposed to this information from coursework or their major.

**Supplementary information:**

**Supplementary information** accompanies this paper at 10.1186/s12889-020-09696-0.

## Background

Mental health literacy—defined as knowledge and beliefs regarding psychological disorders, which in turn fosters the ability to identify, manage, and prevent such disorders—originated in Jorm et al.’s [1] influential paper on this topic. Included in this definition are recognition of the symptoms of psychological disorders, knowledge of their causes and risk factors, attitudes regarding mental health, and the capacity to access both mental health information and professional services. Emerging from this multifaceted construct is the premise that improving the level of mental health literacy within communities and the public at large can lead to early recognition and appropriate intervention for psychological disorders. Due to the high prevalence rates of mental health issues that occur in the college population [2, 3] and because early adulthood is frequently the time of onset for common psychological disorders [4], increasing the mental health literacy of college students is crucial.

Concerns regarding the college population are further established by a recent national survey conducted by the American College Health Association, where when asked about their experiences in the past 12 months, more than 45% of undergraduate students reported having had difficulty functioning due to depression, and more than 65% reported having had overwhelming anxiety [5]. Furthermore, in a recent international study by the World Health Organization, more than 30% of first-year undergraduates reported that at some point in the past 12 months, they experienced at least one of the mood, anxiety, or substance disorders addressed in the survey [6].

With the goal of facilitating better mental health literacy for college students, it is critical to identify the factors related to both increased and decreased knowledge in this area. Doing so provides a unique opportunity to highlight student groups in need of interventions, which when implemented, have the potential to improve mental health literacy in this vulnerable population.

### Demographic and psychological factors associated with mental health literacy

The variable that has most often been studied in relation to mental health literacy is gender, with females repeatedly associated with better mental health literacy. Specifically, females displayed higher rates of recognition than males in studies that have focused on depression [7–10] and anxiety disorders [11], and male gender has been associated with poor mental health literacy in relation to depression [12, 13] and eating disorders [14]. It does seem, however, that gender differences may vary based on disorder being addressed, as a gender difference was apparent for knowledge of depression, but not for knowledge of psychosis [12]. In line with this finding, females perceived a greater need for treatment than did males for both generalized anxiety disorder and psychosis, but no gender difference was observed regarding perceived need for treatment for depression [15]. Furthermore, one study did not report any gender differences in overall mental health literacy [16]. Thus, despite some inconsistencies, overall the literature supports the association of female gender with higher mental health literacy.

In looking at age as a factor associated with mental health literacy, performance of different age groups within studies was compared. One study found that participants in the 18–29 age group displayed higher rates of identification for most anxiety-related disorders as compared to those in the 30–44 and 45–71 age groups [11]. Additionally, another study found that a greater proportion of participants in the youngest age group (20–34 years) showed evidence of depression recognition, as compared to those in the two older age groups (35–49 years and 50–64 years) [7]. Furthermore, in a study on mental health literacy for depression, participants ages 60–69 were determined to have poor cognition in relation to depression when compared to participants ages 30–59 [13]. Similarly, another study determined that participants age 70 and older showed lower ability to recognize symptoms of depression than those in all other age groups (18–24 years; 25–39 years; 40–54 years; 55–69 years) [17]. However, in this same study, those in the youngest group (18–24 years) were more likely than those in the oldest group (70+ years) to incorrectly identify schizophrenia as depression. Challenging the findings that age in general relates to better mental health literacy, no differences were found between those in the 18–24 age group and those in the 25–64 age group in terms of general knowledge of mental health [16]. Therefore, though there seems to be an association between age and mental health literacy, more research is needed in order to establish a clear pattern of findings.

Research has also examined whether experience with mental health-related issues is associated with mental health literacy, with mixed results. In a study that assessed recognition of depression and schizophrenia, previous personal experience with mental health treatment was associated with symptom recognition of these disorders [18]. Furthermore, a study that assessed participants’ ability to identify depression found that having a personal history of treatment for a mental health issue correlated with more positive perceptions about treatment [7]. However, in this same study, neither a personal history of a mental health issue, nor a current episode of depression, was associated with better depression recognition. Adding to these discrepant findings, as the number of psychological diagnoses that participants experienced over the course of their lives increased, and as the number of mental health services being used by their families increased, so did their mental health literacy for mood disorders [19]. This study also found that as the number of current diagnoses of participants increased, knowledge of mood disorders decreased, perhaps suggesting that the presence of current diagnoses negatively impacts mental health literacy. Additionally, severity of a disorder has been found to influence mental health literacy, as one study found that participants categorized as “high” depressed were significantly less likely to recognize depression in comparison to those categorized as “low” depressed [8]. Further complicating the attempt to find an overall relationship between experience with mental health-related issues and mental health literacy, no association was found between personal experience with mental illness and mental health literacy for anxiety disorders [11]. Due to these inconsistent findings, personal experience and its relation to mental health literacy should be further examined.

### Factors associated with mental health literacy related to college experience

Several studies have focused on factors related to mental health literacy in college students [8, 9, 18, 20–23] with some of these studies investigating variables specifically related to college experience, such as year in college and field of study. In line with findings that correct recognition of depression was associated with being in the later years of college study [22], male graduate students were found to have higher mental health literacy than undergraduates [21]. Regarding field of study, participants who had studied psychology and medicine had the highest true symptom scores for both schizophrenia and depression, when compared to students from other disciplines [18], and medically-focused undergraduates were more adept at recognizing depression and knowing about appropriate treatment options [22]. Supporting this finding, participants who had studied psychology or psychiatry reported that they recognized and could define the disorders more often than did students of other fields of study [20]. Thus, evidence seems consistent that both higher years of study and field of study are related to mental health literacy, however, these studies are not common, and additional variables directly related to college experience should be investigated for a more comprehensive understanding of the factors associated with mental health literacy in the college population.

### Current study

Based on a comprehensive review of the literature, we identified several gaps in knowledge related to factors associated with mental health literacy. Some studies included a limited number of variables in their models, and many studies assessed knowledge of just 1–2 disorders, particularly depression and schizophrenia or just depression. Most importantly, however, there are a limited number of studies addressing the factors related to mental health literacy in a college population, few of which include variables related particularly to college experience. The current study seeks to improve upon existing research by: 1) incorporating multifaceted demographic, psychological, and academic variables within a single model, and 2) assessing knowledge and related topics of more than 20 psychological disorders from the *DSM-5* [24]. Some of our included variables have been utilized previously, while others, to our knowledge, are novel and directly relevant to college students’ experiences. Through this comprehensive approach, we seek to capture the variance in performance on an assessment of mental health literacy for college students for the vital purpose of improving knowledge and awareness of mental health in this at-risk population.

## Methods

### Participants and procedure

Data were collected from undergraduate students enrolled at nine different colleges that are part of a large, urban, public university system in the northeastern United States. Using a convenience sampling method, participants were most commonly recruited in classrooms, after members of the research team obtained permission from professors to administer surveys during class time. Other methods of recruitment and administration included in-person invitation in populated campus locations (e.g., cafeterias, student lounges), postings in college-generated subject pool listings, and scheduled administration periods conducted in reserved classrooms. Students were given $5 for their participation, except those from the subject pool who were given research credit. Participants’ multiple choice and handwritten responses were entered into a Statistical Program for Social Sciences (SPSS; [25]) database and each entry was double-checked for accuracy.

A power analysis was conducted using G*Power3 [26], with conservative estimates at 1% significance level, 50% power and a small effect size (f = 0.05). Based on the power analysis (under the assumptions of a MANOVA framework [27]), a total sample size of 300 participants would be required to detect differences in the three performance levels (low, mid-level, high) for mental health literacy scores based on the 11 variables included in the analysis.

This study was designed as a paper-and-pencil survey, which was administered exclusively in-person to prevent participants from searching online for answers to the mental health literacy items. Before taking the survey, the study’s purpose and procedures were read to prospective participants by research assistants, including that the study was about mental health literacy and would take approximately 30–40 min. Prospective participants were also told that participation was voluntary and that they could withdraw at any point without consequence. All methods of recruitment, consent, and administration were conducted according to an IRB-approved protocol.

### Measure

A two-page form preceded the actual survey and inquired about four areas: (1) demographics; (2) college experience; (3) mental health experience; and (4) openness to mental health issues. Participants then answered items from the Mental Health Literacy Assessment for College Students (MHLA-c), which was created by licensed clinical psychologists with expertise in the field of psychopathology and higher education, with some items adapted from the Multiple-Choice Knowledge of Mental Illnesses Test/MC-KOMIT [28]. The MHLA-c is a uni-dimensional instrument, with scores approximately normally distributed, and with preliminary psychometric support including evidence for internal consistency reliability, content validity, and construct validity (refer to [29], for information related to measure development and validation).

To reduce participant burden, as students completed multiple-choice items from the MHLA-c and a two-page form related to demographic and relevant experiential variables, the MHLA-c items were split into two different forms, which each included 38 items (see Additional file 1 for sample items). These items consisted of multiple-choice questions with five possible answer choices, and drew on knowledge and related topics of more than 20 disorders from the *DSM-5* [24]. Content domains included: (1) *knowledge* of mental health disorders including etiology, risk factors, diagnoses, symptoms, treatment, course of illness, and outcome; and (2) *application* of content knowledge including level of insight, manifestation of symptoms in everyday life, responding to others, accessing help from professionals, and prevention of negative outcomes [29].

### Organization of variables

We used percent correct to quantify performance. However, our goal was not to look at individual participant performance, but rather to differentiate between participants who had varying degrees of mental health literacy. Therefore, we categorized participants into low performers (0–32% correct), mid-level performers (33–67% correct), and high performers (68–100% correct) in an attempt to target specific categories of performance. Age was binned into five categories (18–22, 23–27, 28–32, 33–37, and 38+ years) to differentiate the traditional undergraduate college students from those who typically spend more time completing their undergraduate studies or are returning for a college degree. Variables such as gender and ethnicity were scored categorically. Coursework was binned into two categories (presence or absence of a course related to clinical psychology), and included courses such as abnormal psychology, abnormal psychology in children, psychotherapy, and counseling psychology. Current year in college was binned into three categories (first or second year, third year, and fourth year or later) and college major was binned into seven categories (psychology, applied health sciences, STEM [science, technology, engineering, mathematics], humanities/social sciences, business/economics/accounting, education, and other). Responses to experience with psychological disorders were binned on level of exposure (none, some, or more) of personal, family, or peer history with psychological disorders. Finally, personal diagnosis and/or treatment of a psychological disorder was binned into two categories (presence or absence), as was openness of immediate family to talking about mental health issues (yes or no), and consideration of using campus academic and/or mental health services (yes or no).

### Statistical analyses

The purpose of this study was to identify which variables best discriminated between high, mid-level, and low performers on an assessment of mental health literacy. As our research question was correlational rather than predictive in nature, and our data were a combination of categorical, numeric, and ordinal variables, we used a discriminant correspondence analysis or DiCA [30, 31], which preserves the inherent categorical nature of these multivariate data as opposed to a traditional discriminant analysis or logistic regression. DiCA is an extension of Correspondence Analysis and Multiple Correspondence Analysis [32, 33], and these techniques handle categorical data in the same way that discriminant analysis and principal components analysis handle continuous data [30]. DiCA analyzes the differences between categories of observations (e.g., performance levels) based on multiple variables (e.g., age, gender, field of study), and represents these differences in the form of new, uncorrelated variables known as components, which are linear combinations of the original variables. These components reveal how categories of observations (e.g., performance levels) are different from each other, and which variables (e.g., age, gender, field of study) contribute to those differences. For a particular component, categories that are dissimilar to each other are oppositely signed, and categories that are similar to each other have the same sign (see [34] for a more detailed application of DiCA).

In the current study, DiCA was used to identify qualitative differences between patterns of responses on mental health literacy scores based on demographics (age, gender, ethnicity), college experience (year in college, college major, coursework), mental health experience (having been diagnosed and/or treated for a psychological disorder, having experience based on personal, family, or peer history with a psychological disorder), and openness to mental health issues (considering the use of academic and/or mental health college services, openness of family to discuss mental health issues).

### Inference procedures

For DiCA, a permutation test is used to determine whether the overall variance of the data is statistically significant, and to also determine whether the variance explained by each component is statistically significant [35]. In addition, bootstrap tests are used to generate multivariate confidence intervals to differentiate between categories in the overall component space, and to also identify which variables significantly contribute to each component [31, 34, 36–39] (see [34] for more details on inference procedures). While data organization was performed on SPSS and Microsoft Excel (2011), all further statistical analyses were conducted in R [40] as DiCA was specifically created using the R programming language [41]. Tables for descriptive statistics were generated using jamovi (also an R-based software; [42]).

## Results

### Descriptive findings

Data were collected from 1255 participants, but due to missing data, 42 participants were excluded from the final statistical analysis, which resulted in a final sample size of 1213 participants. Specifically, 18 participants omitted the questions on diagnosis and/or treatment; 14 omitted their age; 6 omitted their year in college; 2 omitted their ethnicity; 1 omitted gender; and 1 omitted both questions on age and diagnosis and/or treatment.

The final set of variables, their levels, and a summary of the descriptive statistics can be found in Table 1. Mental health literacy scores were approximately normally distributed and categorized as follows: 18.2% fell into the 0 – 32nd percentile (low); 53.5% fell into the 33 – 67th percentile (medium); and 28.3% fell into the 68 – 100th percentile (high).

**Table 1:** Demographic variables (*n* = 1213)

Variables (Number of Levels)	% (n)
**Age (5)**	
*18–22*	68.9 (836)
*23–27*	20.9 (253)
*28–32*	5.4 (66)
*33–37*	2.7 (33)
*38+*	2.1 (25)
**Gender (3)**	
*Female*	62.0 (752)
*Male*	37.5 (455)
*Other*	0.5 (6)
**Race (6)**	
*Black/African American*	27.3 (331)
*White/Caucasian*	26.7 (324)
*Asian/Asian American*	19.0 (231)
*Hispanic/Latino*	18.5 (224)
*Multiracial*	5.9 (72)
*Native American*	0.3 (4)
*Other*	2.2 (27)
**Year in College (3)**	
*First-Second*	39.3 (477)
*Third*	31.1 (377)
*Four+*	29.6 (359)
**College Major (7)**	
*Psychology*	26.7 (324)
*STEM*	21.5 (261)
*Humanities/Social Sciences*	16.0 (194)
*Applied Health Science*	15.3 (186)
*Business/Economics/Accounting*	14.0 (170)
*Education*	4.2 (51)
*Other*	2.2 (27)
**Clinical Psychology Course (2)**	
*Yes*	25.0 (303)
*No*	75.0 (910)
**Experience with Psychological Disorders (3)**	
*None*	31.6 (383)
*Some*	61.1 (741)
*More*	7.3 (89)
**Diagnosed/Treated (2)**	
*Yes*	14.2 (170)
*No*	86.0 (1043)
**Family Openness to Discussion (2)**	
*Yes*	53.8 (652)
*No*	46.2 (561)
**Academic Services (2)**	
*Yes*	84.3 (1023)
*No*	15.7 (190)
**Mental Health Services (2)**	
*Yes*	74.7 (906)
*No*	25.3 (307)
**Percentage Score (3)**	
*Low*	18.2 (221)
*Medium*	53.5 (649)
*High*	28.3 (343)

### DiCA findings

**Fig. 1:**
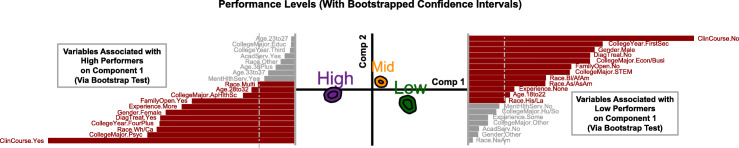
Results from DiCA showing bootstrap confidence intervals for three performance levels (center), and bootstrap ratio bars for statistically significant variables associated with high performers (left) and low performers (right) for component 1. Longer bars represent more reliable variables associated with performance, while shorter bars represent statistically significant, but less reliable variables associated with performance. The dotted vertical lines represent a *p* < 0.05 threshold such that variables that do not cross the dotted lines (shown in light grey) are not statistically significant for that component

DiCA generated two components that described the overall variance of the data. Component 1 represented the difference between high-performers and low performers, while component 2 represented the difference between the mid-level performers and all other performers (Fig. 1, center panel). The overall variance (also known as *inertia*) was found to be statistically significant via a permutation test (inertia = 0.049, *p*_*perm*_ < 0.001). The variance explained by each component was also statistically significant (component 1 = 90.27%, *p*_*perm*_ < 0.001, component 2 = 9.73%, *p*_*perm*_ < 0.001), via separate permutation tests. The reliability of assignment of individuals to their respective performance categories (low, mid-level, high) was also found to be statistically significant (*R*^2^ = 0.18, *p*_*perm*_ < 0.001). Finally, bootstrap ratio tests showed that low, mid-level, and high performers statistically differed from each other (*p*_boot_ < 0.001) and contributed to the overall variance of the data (Table 2).

**Table 2:** Component scores **(bootstrap ratios)** for each grade category (*n* = 1213)

Mental Health Literacy Performance Category	Component 1	Component 2
*Low*	0.28 **(13.73)**^a^	− 0.11 **(−5.04)**^a^
*Medium*	0.07 **(4.96)**^a^	0.06 **(5.3)**^a^
*High*	−0.31 **(−14.08)**^a^	− 0.04 **(−2.39)**^a^

^a^ Bootstrap ratios above/below +/− 1.96 are considered significant

These findings imply that overall there exists a statistically significant difference in the levels of mental health literacy performance across participants. Specifically, the largest variance in the data was explained by the difference in the pattern of responses of low performers as compared to high performers, followed by the pattern of responses of mid-level performers.

In order to determine which variables significantly contributed to the variance explained by both component 1 and component 2, additional bootstrap tests were conducted for each variable (Table 3). The variables that significantly contributed to the difference between the high and low performers were: coursework related to clinical psychology, college major, diagnosis and/or treatment for a psychological disorder, ethnicity, year in college, gender, experience with psychological disorders based on personal, family or peer history, family openness to discussing mental health issues, and age (Fig. 1, right and left panels).

**Table 3:** Component scores **(bootstrap ratios)** for every level for each variable (*n* = 1213)

Variables (Number of Levels)	Component 1	Component 2
**Age (5)**		
*18–22*	0.04 **(2.24)**^a^	0.00 **(0.20)**
*23–27*	− 0.01 **(− 0.19)**	− 0.01 **(− 0.15)**
*28–32*	− 0.29 **(− 2.36)**^a^	− 0.03 **(− 0.28)**
*33–37*	− 0.26 **(− 1.45)**	− 0.01 **(− 0.04)**
*38+*	− 0.20 **(− 1.04)**	0.04 **(0.25)**
**Gender (3)**		
*Female*	−0.15 **(−7.04)**^a^	0.08 **(3.44)**^a^
*Male*	0.25 **(6.87)**^a^	−0.13 **(−3.51)**^a^
*Other*	0.19 **(0.51)**	0.21 **(0.51)**
**Race (6)**		
*Black/African American*	0.18 **(4.21)**^a^	0.04 **(0.88)**
*White/Caucasian*	−0.37 **(−7.53)**^a^	−0.09 **(−2.10)**^a^
*Asian/Asian American*	0.23 **(4.17)**^a^	0.06 **(0.98)**
*Hispanic/Latino*	0.11 **(2.00)**^a^	0.02 **(0.26)**
*Multiracial*	−0.23 **(−2.02)**^a^	0.10 **(0.98)**
*Native American*	0.13 **(0.22)**	−0.12 **(− 0.18)**
*Other*	−0.18 **(− 0.77)**	− 0.28 **(−1.55)**
**Year in College (3)**		
*First-Second*	0.25 **(7.30)**^a^	−0.11 **(−3.01)**^a^
*Third*	−0.01 **(− 0.26)**	0.07 **(1.64)**
*Four+*	−0.32 **(−7.37)**^a^	0.07 **(1.70)**
**College Major (7)**		
*Psychology*	−0.4 **(−8.04)**^a^	0.01 **(0.17)**
*STEM*	0.28 **(5.51)**^a^	−0.07 **(−1.22)**
*Humanities/Social Sciences*	0.10 **(1.50)**	0.00 **(−0.03)**
*Applied Health Science*	−0.19 **(−2.82)**^a^	0.02 **(0.35)**
*Business/Economics/Accounting*	0.40 **(6.60)**^a^	−0.05 **(− 0.62)**
*Education*	−0.02 **(− 0.20)**	0.33 **(2.78)**^a^
*Other*	0.18 **(1.05)**	0.14 **(0.68)**
**Clinical Psychology Course (2)**		
*Yes*	−0.63 **(−13.45)**^a^	−0.09 **(−2.3)**^a^
*No*	0.21 **(12.28)**^a^	0.03 **(2.32)**^a^
**Experience with Psychological Disorders (3)**		
*None*	0.10 **(2.49)**^a^	0.07 **(1.63)**
*Some*	0.02 **(1.05)**	−0.02 **(−0.89)**
*More*	−0.62 **(−6.17)**^a^	−0.12 **(−1.50)**
**Diagnosed/Treated (2)**		
*Yes*	−0.55 **(−7.34)**^a^	−0.13 **(−2.2)**^a^
*No*	0.09 **(6.61)**^a^	0.02 **(2.19)**^a^
**Family Openness to Discussion (2)**		
*Yes*	−0.14 **(−5.61)**^a^	0.03 **(1.15)**
*No*	0.16 **(5.58)**^a^	−0.04 **(−1.15)**
**Academic Services (2)**		
*Yes*	−0.01 **(− 0.55)**	0.05 **(3.70)**^a^
*No*	0.04 **(0.55)**	−0.26 **(−3.83)**^a^
**Mental Health Services (2)**		
*Yes*	−0.03 **(−1.66)**	0.02 **(1.07)**
*No*	0.08 **(1.66)**	−0.05 **(−1.07)**

^a^ Bootstrap ratios above/below +/−1.96 are considered significant

Component 1 specifically revealed that high-performers were more likely to have taken at least one course related to clinical psychology, to typically major in psychology and applied health science fields, and to currently be in the fourth year or higher of their undergraduate program. These participants were also more likely to report being female, white, and between the ages of 28–32. In addition, high-performers were more likely to have been diagnosed and/or treated for a psychological disorder, to have more experience with psychological disorders based on personal, family, or peer history, and to have families who are reported to be more open to discussing mental health issues.

In contrast, low-performers were less likely to have taken a clinical psychology course, to typically major in economics/business or STEM fields, and to currently be in the first or second year of their undergraduate program. These participants were also more likely to report being male, Asian/Asian American, Black/African American, or Hispanic/Latino, and between the ages of 18–22. In addition, low-performers were less likely to have been diagnosed and/or treated for a psychological disorder, less likely to have experience with psychological disorders through personal, family, or peer history, and less likely to have families who were reported to being open to discussing mental health issues.

Component 2 identified the mid-level performers as being different from high or low-level performers. This difference was driven by participants who were more likely to be female, to major in education, to have not taken any clinical psychology course, to have not been diagnosed and/or treated for a psychological disorder, but who were more likely to consider taking campus-offered academic services.

## Discussion

The present study sought to identify factors associated with mental health literacy in a diverse group of undergraduate students. Mental health literacy was quantified using multiple-choice items that assessed conceptual knowledge of specific disorders and the application of that knowledge in everyday life.

We used Discriminant Correspondence Analysis (DiCA), which is a versatile technique for analyzing multiple variables within a single model. This technique is novel and has, thus far, not been used in studies that examine the factors associated with mental health literacy. Using DiCA, we identified student groups who had higher mental health literacy scores. However, in light of the purpose of the study, which was to understand the mental health needs of college students, it was vital to also focus on those student groups with *lower* mental health literacy scores. In highlighting these results, we shed light on the vulnerable student groups in need of intervention for the purpose of increasing mental health literacy in a college population.

### Component 1 findings

The main contributor to variation in scores between high, mid-level, and low performers was having taken a course related to clinical psychology. This finding, though correlational, suggests that formal coursework related to clinical psychology positively affects literacy of mental health. Though previous research has not specifically investigated whether clinical coursework for college students directly increases mental health literacy, there is evidence that *Mental Health First Aid/MHFA* [43], an educational training program where participants are trained to help others in crises related to mental health, improved participants’ mental health knowledge, recognition of psychological disorders, and knowledge of effective treatments [44–46]. Additionally, *Transitions* [47, 48], an educational resource for post-secondary students, which addresses life-skills and mental health information, improved students’ knowledge of mental health, decreased stigma, and increased help-seeking behaviors [49, 50]. Evidence that these programs have had a positive impact on mental health literacy of participants underscores the importance and potential benefits of education in this area.

The question of whether mental health literacy can be taught as a course is an important one. Underlying our main finding, where taking a class related to clinical psychology impacted mental health literacy, is the following question: Is the clinical psychology class in itself incorporating literacy of mental health and thus increasing students’ scores on an assessment of mental health literacy? Or, do students who have higher mental health literacy to begin with, gravitate towards these types of classes? If the former, then the argument can be made that mental health literacy could be taught, but if the latter, would taking such a course actually be effective in increasing mental health literacy? More research is needed to answer this question, specifically to assess if a college course focusing on mental health would increase the mental health literacy of students who have not taken a class related to clinical psychology.

Another factor accounting for the difference in scores between high and low performers was majoring in psychology and applied health science fields, as compared to majoring in other fields, specifically business/economics or STEM fields. This finding corresponds to a study that reported that students of psychology and medicine displayed a higher level of mental health literacy, as well as having determined that male students of natural science, economics, and law were particularly weak at recognizing symptoms of schizophrenia and depression [18]. Further supporting this result is a finding that male STEM majors had lower mental health knowledge than students from non-STEM fields [21]. In general, these studies have examined the relationship between overall disciplines and mental health literacy as opposed to individual majors, and our study, as well, assessed domains of study as opposed to particular majors. However, if participants would be studied more narrowly, via their specific majors, more information could be provided on how concentrated areas of study relate to mental health literacy. Thus, further research is needed to investigate whether differences observed in mental health literacy performance are associated with any individual college majors, with the purpose of directing interventions towards these specific groups.

All of the demographic variables including gender, age, and ethnicity, significantly contributed to differences in mental health literacy scores. Specifically, students who reported being female, white, and between the ages of 28–32, were more likely to earn higher scores as compared to students who reported being male, Asian/Asian American, Black/African American, or Hispanic/Latino, and between the ages of 18–22 years. Our finding that females tend to score higher than males aligns with the literature on gender and mental health literacy in college settings [51]. These consistent findings may allude to the premise that gender socialization is at the core of the apparent gender discrepancies of mental health literacy (see [51] for a discussion on gender socialization and how it relates to mental health literacy).

Participants in the 28–32 age group were more likely to be among the high performers, while participants in the 18–22 age group were more likely to be among the low performers. This finding seems to differ from previous research that found that individuals in the youngest age groups scored highest on identification of disorders [7, 11]. However, there is, in fact, agreement between our results and these studies because the ages of our highest scoring group (28–32) aligns with the upper ages of the youngest groups (18–29 and 20–34) in these studies. Also noteworthy is that participants in our study who had the highest scores were older within a relatively young age group, which parallels a study addressing age and mental health literacy, where being older, albeit within a relatively young age group, was associated with higher performance in university students [9]. However, our results are difficult to directly compare with previous studies due to the variation in age groups. For example, other studies’ oldest age groups were 60–69 [13] and 70+ [17] and our oldest age group was 38+, with only 7 participants above the age of 50. Similarly, it is difficult to compare the results of our lowest scoring group (18–22) with other studies, as research on this age bracket in relation to mental health literacy is scarce. This is unfortunate because the traditional age of college students falls approximately in this age bracket and based on our results these may be the students who are most in need of intervention. In future research, greater consistency in the age ranges utilized across similar samples would help reveal the true pattern of relationship between age and mental health literacy.

In terms of ethnicity, our finding corresponds to a study that found that students who were white had higher scores on depression recognition, as compared to students who were non-white [8]. In further support, a study on college-age males found that undergraduate students who were white had higher mental health literacy than Asian and other undergraduates [21]. It has been suggested that these results may be the effect of mental health literacy reflecting a Western conceptualization of mental health (see [52] for a discussion on mental health literacy as it relates to cultural diversity), possibly calling into question the overall conclusion that non-whites have lower mental health literacy than whites. With this in mind, mental health assessments should incorporate more culturally aligned items in order to tap into experiences of minorities regarding knowledge, awareness, attitude, and treatment of mental health.

In our sample, students who were in their fourth year or later of their undergraduate program scored higher than students in their first, second, or third year. Though this may be the result of increased academic knowledge and life experience, it may also be that familiarity with a college campus makes it more likely for a student to access available mental health services, a factor that potentially contributes to increased mental health literacy.

Having been diagnosed and/or treated for a psychological disorder impacted mental health literacy performance in our sample. Though there is limited research on whether having been diagnosed affects mental health literacy in college students, treatment experience has been shown to impact symptom recognition of depression and schizophrenia [18] and generalized anxiety disorder [8]. In the general population, however, some studies have found that being diagnosed or treated for a mental health issue does influence knowledge of certain mental health disorders [53], while others have found that it does not [7, 11, 54]. Though research has not established a consensus, our findings were, nonetheless, statistically significant. The inconsistency in results may be related to our population of study and may suggest that being diagnosed or treated impacts mental health literacy, particularly in college students. More research is needed to determine if this is so, with the possibility that the significance of this factor varies based on the population being studied. Another possibility is that personal experience with mental health issues is broader than has been addressed in previous research. Rather than personal experience being limited to personal diagnosis and/or treatment or general use of mental health services, we also extended experience with psychological disorders to include one’s family or close friends. These items were included in a question that asked respondents to check off as many areas of experience that pertained to them. Results were statistically significant and, in fact, the more experience respondents reported to have had, the more likely they were to have higher scores.

The role of family is important for an individual’s well-being, especially in the area of mental health. Prior research has found that respondents regard family as an important source of help for mental health issues [10, 55], though family openness to discussing mental health issues and its impact on mental health literacy does not seem to have been addressed. In an attempt to investigate the association between these two variables, we asked respondents if their immediate family was open to talking about mental health issues and those who responded in the affirmative were more likely to have higher mental health literacy scores. This finding suggests that openness to discussing mental health issues may play a role in the mental health literacy of college students. It is interesting to note that in contrast to other variables investigated in this study such as gender, age, ethnicity, year in college, and being diagnosed and/or treated for a psychological disorder, this variable, much like the clinical course previously discussed, is not immutable and can thus be incorporated into an intervention. Doing so as a community outreach initiative or family training would have the potential to increase mental health literacy in a meaningful and far-reaching manner.

The variable that did not have a significant impact on differentiating between low, mid-level, and high-performing participants was potential use of college services. Specifically, we asked respondents whether they would consider taking advantage of various campus mental health services (e.g., personal counseling, drug and alcohol counseling, and mental health awareness training) and academic services (e.g., time management, stress management, test anxiety management), to which they answered “yes” or “no”. It is possible that our non-significant findings relate to the way in which we phrased the question. As opposed to asking about willingness to access campus services, a better query might have been a measure of treatment use, such as whether participants had actually accessed any campus services, as treatment utilization behaviors have been associated with higher mental health literacy [56].

### Component 2 findings

Based on results from component 2, one of the statistically significant variables that separated mid-level performers from high and low-level performers was being an education major. Specifically, mid-level performers were more likely to be female, to be education majors, to have not taken a clinical course, to not have been diagnosed and/or treated, but who would consider using academic services in areas such as test anxiety, stress management, and time management. This might relate to the premise that education majors have more of an awareness of mental health-related issues, as compared to STEM or business/economics majors. Furthermore, the willingness of these education majors to consider campus-offered academic services, a variable found to be statistically significant for component 2, but not statistically significant for component 1, may relate to the value that education majors place on educational services.

### Study limitations and future directions

Our study has the strength of assessing mental health literacy in a large and diverse sample of undergraduate college students utilizing numerous variables, which to our knowledge are more extensive than have been previously incorporated in a single study. However, due to the study design and logistical issues, we were not able to randomly select students for participation and instead used a convenience sample. In addition, as participation was voluntary, we had a higher percentage (62.0%) of women in our sample. Furthermore, all participants were from the same city and enrolled at commuter colleges, a population that is quite different from traditional undergraduates. In light of this, it is difficult to ascertain whether findings can be generalized to undergraduate students from a different geographic location and enrolled at a residential college. Also, as noted above, this study was correlational and conclusions about the directionality of the findings cannot be drawn—particularly for variables such as college major and formal coursework related to clinical psychology.

In terms of future directions, efforts should focus on addressing vulnerable students’ mental health needs by: 1) increasing awareness of, and access to, clinical services available on campus, especially to those who typically do not feel comfortable availing themselves to such services, such as males and minority groups, in a manner that is culturally accommodating and sensitive; and 2) developing an educational curriculum intended to increase mental health literacy across majors and offering a 1-credit abnormal psychology “light” course to students during their freshman and sophomore years in college. Though important in terms of its broad sweep as an educational intervention, ideally, it is the student groups with lower mental health literacy performance that would be targeted for this course, where data collected in this area could then foster a campaign geared towards these students and provide a rationale for intervention (e.g., providing psychoeducation, promoting awareness of college resources, increasing availability of treatment), all at the college level.

## Conclusion

Due to the prevalence of psychological disorders among the college population, students’ mental health literacy, which includes understanding of mental health disorders and how to recognize, manage, and seek treatment for such disorders, is critical. In our study, the most robust contributors to mental health literacy were: 1) *coursework*: those who have taken a clinical psychology course, particularly those who are psychology majors and; 2) *experience*: those who have experience with mental health issues because they have been diagnosed and/or treated for a psychological disorder or because they have family or experience with a psychological disorder. Our findings offer a basis for understanding the mental health needs of diverse undergraduate students by providing an opportunity to identify not only those with high mental health literacy, but also those with low mental health literacy. Identification of both of these groups is critical in providing a direction for intervention in terms of educational and clinical services, with the aim of increasing the mental health literacy and overall psychological well-being of college students.

## Supplementary information

**Additional file 1.** The items in Additional file 1 are multiple-choice questions that are similar in content and structure to items on the MHLA-c.

## Data Availability

The datasets used for the current study are available from the corresponding author upon reasonable request. Additionally, the survey will be made available to interested researchers.

## Abbreviations

DiCA: Discriminant Correspondence Analysis
DSM: Diagnostic and statistical manual of mental disorders
MHLA-c: Mental Health Literacy Assessment for College Students
STEM: Science, technology, engineering, and mathematics
