# Supplementary material for: Mental health literacy in a diverse sample of undergraduate students: demographic, psychological, and academic correlates
Source: BMC Public Health. 2020 Nov 13;20:1699. doi: 10.1186/s12889-020-09696-0 (PMC7663887; doi:10.1186/s12889-020-09696-0)
Supplement: Supplementary file 1 — Additional file 1. The items in Additional file 1 are multiple-choice questions that are similar in content and structure to items on the MHLA-c. [file 12889_2020_9696_MOESM1_ESM.docx]

**Additional file 1**

Sample items similar to the items on the MHLA-c:

1) Your friend is a first-year college student who for years has experienced anxiety before making a presentation in front of the class. She tells you that for days before presenting she experiences dizziness, fast heartbeat, difficulty sleeping, and she cannot stop thinking about what her classmates and instructor will think of her and the possibility that they will judge her. Your friend would most likely be diagnosed with:

1. panic disorder
2. somatoform disorder
3. social anxiety disorder
4. generalized anxiety disorder
5. obsessive-compulsive disorder

2) Seizures may occur during withdrawal from heavy, long-term use of which substance?

1. alcohol
2. diet pills
3. marijuana
4. mushrooms
5. nicotine

3) Which of the following is an effective treatment for post-traumatic stress disorder (PTSD)?

1. psychoanalysis
2. electroconvulsive therapy
3. hormone therapy
4. cognitive behavioral therapy
5. social skills training

4) Your college roommate has been struggling with sadness, anxiety, and thoughts of hurting himself, and he has agreed to seek help. Each of the following professionals could provide effective treatment except:

1. a social worker
2. a clinical psychologist
3. a psychiatrist
4. a mental health counselor
5. a research psychologist

5) You're worried about your health, your family, bills, and schoolwork. How can you determine if this anxiety is normal or abnormal?

1. the presence of physical symptoms
2. the presence of fear
3. an effect on daily functioning
4. a change in self-esteem
5. a change in appetite
